# The role of tRNA identity elements in aminoacyl-tRNA editing

**DOI:** 10.3389/fmicb.2024.1437528

**Published:** 2024-07-18

**Authors:** Edwin Cruz, Oscar Vargas-Rodriguez

**Affiliations:** Department of Molecular Biology and Biophysics, University of Connecticut School of Medicine, Farmington, CT, United States

**Keywords:** tRNA, mistranslation, translational fidelity, protein synthesis, aminoacyl-tRNA synthetases, editing

## Abstract

The rules of the genetic code are implemented by the unique features that define the amino acid identity of each transfer RNA (tRNA). These features, known as “identity elements,” mark tRNAs for recognition by aminoacyl-tRNA synthetases (ARSs), the enzymes responsible for ligating amino acids to tRNAs. While tRNA identity elements enable stringent substrate selectivity of ARSs, these enzymes are prone to errors during amino acid selection, leading to the synthesis of incorrect aminoacyl-tRNAs that jeopardize the fidelity of protein synthesis. Many error-prone ARSs have evolved specialized domains that hydrolyze incorrectly synthesized aminoacyl-tRNAs. These domains, known as editing domains, also exist as free-standing enzymes and, together with ARSs, safeguard protein synthesis fidelity. Here, we discuss how the same identity elements that define tRNA aminoacylation play an integral role in aminoacyl-tRNA editing, synergistically ensuring the correct translation of genetic information into proteins. Moreover, we review the distinct strategies of tRNA selection used by editing enzymes and ARSs to avoid undesired hydrolysis of correctly aminoacylated tRNAs.

## 1 Introduction

**FIGURE 1:**
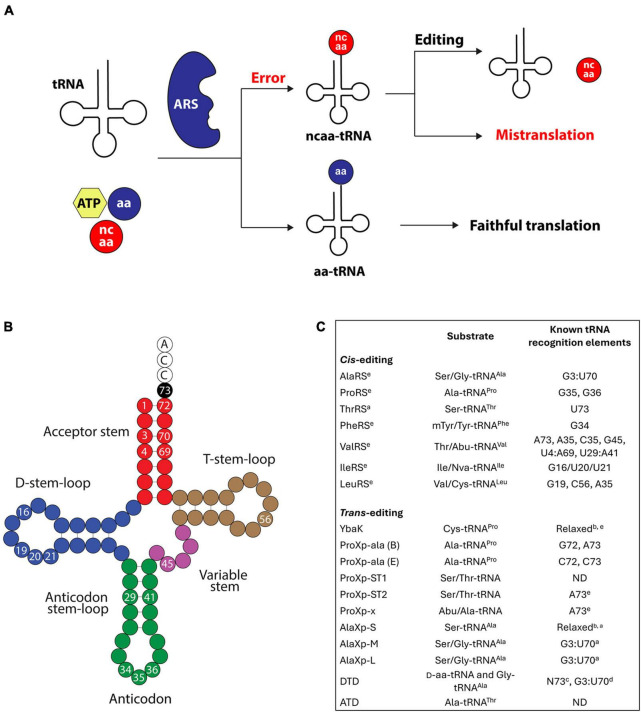
**(A)** Steps in tRNA aminoacylation and editing. tRNAs are aminoacylated by ARSs producing aa-tRNAs. If the ARS uses a non-cognate amino acid (ncaa), the resulting ncaa-tRNA can be hydrolyzed by the editing enzymes. In the absence of editing checkpoints, the ncaa is incorporated into proteins in response to the wrong codon, causing mistranslation. **(B)** Representative secondary structures of tRNAs. As discussed in the main text, the numbered bases indicate the various positions important for editing. **(C)** Summary of the *trans*- and *cis*-editing domains with characterized functions and their known tRNA recognition elements. ^a^Archaeal origin; ^b^indicates weak or no tRNA specificity; ^c^the specificity of N73 depends on the DTD’s origin; ^d^in the context of tRNA^Ala^; ^e^Bacterial origin; “ND” indicates not determined. B and E for ProXp-Ala indicate bacterial and eukaryotic, respectively.

Accurate translation of mRNAs into proteins requires the correct synthesis of aminoacyl-tRNAs (aa-tRNAs). This reaction, known as tRNA aminoacylation or charging, is catalyzed by aminoacyl-tRNA synthetases (ARSs), which ligate amino acids to tRNAs (Ibba and Soll, 2000). Errors in amino acid or tRNA selection by ARSs lead to incorrectly synthesized aa-tRNAs (Figure 1A). Generally, ARSs display a more robust specificity for their tRNA substrates than for amino acids. The relatively weaker amino acid specificity is mainly due to the structural and chemical similarities shared by many proteinogenic and non-proteinogenic amino acids (Ling et al., 2009a; Bullwinkle et al., 2014a; Hoffman et al., 2017; Mohler and Ibba, 2017). As a result, many ARSs do not effectively discern between cognate and near-cognate amino acid substrates. Prominent examples of tRNA mischarging include threonyl-tRNA synthetase (ThrRS), which confuses Ser for Thr (Dock-Bregeon et al., 2000), and isoleucyl-tRNA synthetase (IleRS), which mistakes Val for Ile (Berg et al., 1961). If uncorrected, tRNA aminoacylation errors lead to the translation of codons with the wrong amino acid (mistranslation), which can cause cellular dysregulation, growth defects, and death (Lee et al., 2006; Nangle et al., 2006; Ling and Soll, 2010; Bullwinkle et al., 2014b; Cvetesic et al., 2014; Liu et al., 2014, 2015; Lu et al., 2014; Kelly et al., 2019; Lant et al., 2019; Zhang et al., 2021; Schuntermann et al., 2023).

Due to their propensity to charge tRNAs with the wrong amino acid, ARSs acquired specialized hydrolytic domains to “edit” their aa-tRNA products. These domains, known as “editing” domains, catalyze the hydrolysis of mischarged tRNAs, ensuring that only correctly aminoacylated tRNAs accumulate in the cell (Figure 1A). In addition to the editing domains embedded in ARSs (known as *cis*-editing domains), aa-tRNA hydrolysis is catalyzed by standalone deacylases (known as *trans*-editing domains) (Kuzmishin Nagy et al., 2020; Jani and Pappachan, 2022). *Cis*- and *trans*-editing domains act as essential quality control checkpoints to maintain the integrity of the genetic code. The importance of aa-tRNA editing is underscored by the negative phenotypes associated with defects in editing domains (Lee et al., 2006; Nangle et al., 2006; Ling and Soll, 2010; Bullwinkle et al., 2014b; Cvetesic et al., 2014; Liu et al., 2014, 2015; Lu et al., 2014; Kelly et al., 2019; Lant et al., 2019; Zhang et al., 2021).

In contrast to amino acids, ARSs identify their tRNA substrates through an intricate set of structural and sequence features unique to each tRNA (Schimmel et al., 1993; Giege et al., 1998; Giege and Eriani, 2023). These tRNA features, collectively known as identity elements, promote faithful interactions between tRNAs and ARSs, preventing ARSs from cross-reacting with non-cognate tRNAs. Notably, growing evidence indicates that many editing domains rely on the same tRNA elements to gain aa-tRNA specificity and avoid hydrolysis of correctly aminoacylated tRNAs. This tRNA specificity is crucial to elude unintended energy loss due to the depletion of correctly aminoacylated tRNAs and to maintain adequate aa-tRNA supply for protein synthesis. More importantly, the role of tRNA identity elements in aa-tRNA editing highlights how identity elements secure the accurate translation of the genetic code.

## 2 tRNA identities

The elements that define the identity of tRNAs for a particular amino acid primarily reside in the tRNA acceptor stem and the anticodon loop (Figure 1B; Giege et al., 1998; Beuning and Musier-Forsyth, 1999; Giege and Eriani, 2023). Positions 1, 72, and 73 in the acceptor stem, and 35 and 36 in the anticodon are major contributors to tRNA selection. These elements act as an operational code to mark tRNAs for aminoacylation by a specific ARS (Schimmel et al., 1993; Ribas de Pouplana and Schimmel, 2001). Identity elements in the acceptor stem are generally recognized in the aminoacylation site of ARSs, whereas dedicated anticodon binding domains mediate the recognition of tRNA anticodon elements. tRNA identity elements are typically conserved within a single domain of life. However, with few exceptions, they diverge across domains of life (Lin et al., 2019). For example, the operational code for aminoacylation of tRNA^Pro^ diverged during evolution from G72 and A73 in bacteria to C72/A73 and C72/C73 in archaea and eukaryotes, respectively (Liu et al., 1995; Stehlin et al., 1998; Burke et al., 2001). These changes in tRNA^Pro^ were accompanied by changes in the selection mechanism of prolyl-tRNA synthetase (ProRS), preventing cross-reaction between ProRS and tRNA^Pro^ from different domains of life (Stehlin et al., 1998; Burke et al., 2001). Similar changes in the operational code of other tRNAs are known (Giege and Eriani, 2023).

## 3 The diversity of editing

**FIGURE 2:**
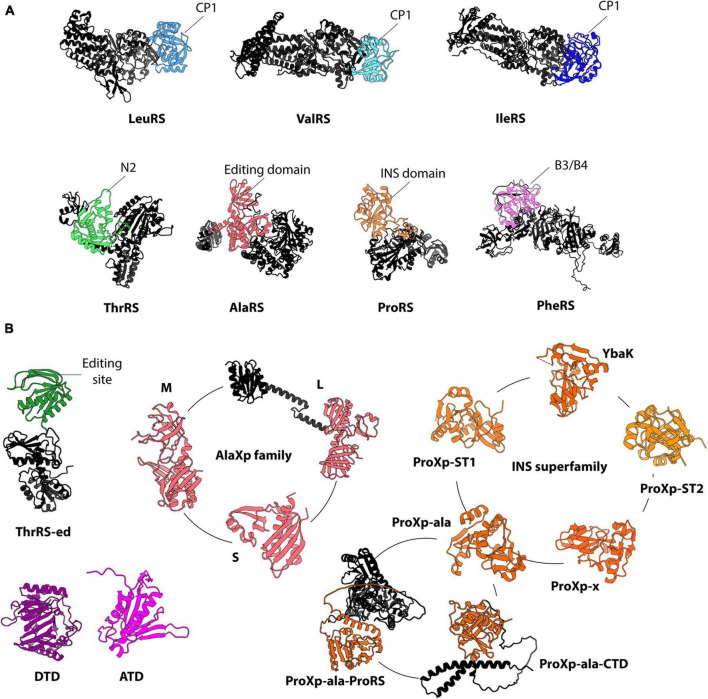
Representative structures of ARSs’ editing domains **(A)** and free-standing editing enzymes **(B)**. The CP1 domains of LeuRS (PDB 3ZJU), ValRS (PDB 1IVS), and IleRS (PDB 1FFY) are colored in light blue, teal, and navy blue, respectively. The editing domains of ThrRS (PDB 1NYQ), AlaRS (PDB 3WQY), ProRS (PDB 2J3L), and PheRS (PDB 3PCO) are shown in green, pink, orange, and purple, respectively. The other domains (e.g., aminoacylation and anticodon binding domains) are in black. For ThrRS-ed (an AlphaFold model of *S*. *solfataricus*), the hydrolytic active domain is shown in green, while the anticodon binding domain is in black. The structure of *E*. *coli* DTD (PDB 1JKE) and the AlphaFold model of human ATD are shown. The INS superfamily is represented by ProRS and five single-domain families: YbaK (PDB 1DBU), ProXp-ST1 (an AlphaFold model of *E*. *coli*), ProXp-ST2 (an AlphaFold model of *Bordetella parapertussis*), ProXp-ala (PDB 5VXB), ProXp-ala-CTD (an AlphaFold model of *Arabidopsis thaliana*), ProXp-ala-ProRS (an AlphaFold model of *Plasmodium falciparum*) and ProXp-x (PDB 2CX5). ProXp-7, ProXp-8, and ProXp-9 were omitted from the INS superfamily because their activities are unknown. The three known isoforms of AlaXp are represented by the structures of AlaXp-S (PDB 1WXO), AlaXp-M (PDB 2E1B), and AlaXp-L (an AlphaFold model of *Pyrococcus horikoshii*). For simplicity, all structures are displayed in monomeric form.

Seven ARS families have editing domains to proofread aa-tRNA synthesis, whereas five families and superfamilies of *trans*-editing domains are currently known (Figure 2; Kuzmishin Nagy et al., 2020; Jani and Pappachan, 2022). In most cases, *trans*-editing domains are evolutionarily related to the editing domains of ARSs, sharing structural homology and, sometimes, substrate specificity. *Trans*- and *cis*-editing domains employ diverse mechanisms of substrate selection, which can involve unique characteristics of the amino acid side chain or tRNA features. Most editing domains use steric exclusion and/or chemical mechanisms to differentiate aminoacyl moieties of aa-tRNAs. Consequently, they tend to display relaxed amino acid specificities. For example, bacterial ProXp-ala, a *trans*-editing domain, hydrolyzes Ala- and Ser-tRNA with similar efficiency (Danhart et al., 2017). In contrast to their aminoacyl moiety selectivity, both *trans*- and *cis*-editing domains, with some exceptions, exhibit more robust tRNA specificities. The tRNA selectivity of editing enzymes can be mediated via direct or indirect interactions. These mechanisms of tRNA recognition are discussed in the following section.

## 4 Identity elements in aminoacyl-tRNA editing

Accurate recognition of mischarged tRNAs by editing enzymes is essential to avoid deacylation of correctly aminoacylated tRNAs. Because aa-tRNA synthesis requires an ATP molecule, indiscriminate hydrolysis of correctly charged tRNA by editing enzymes would be energetically costly and could impact cell growth and homeostasis by decreasing the available pool of aa-tRNAs for protein synthesis. As discussed in the following subsections, editing domains have evolved distinct mechanisms of substrate selection that ensure hydrolysis of the incorrect aa-tRNAs. Notably, in many cases, the same tRNA identity elements that define aminoacylation are used to gain specificity during editing (Figure 1C). However, lacking tRNA specificity in other cases may offer a functional advantage in acting on diverse mischarged tRNA substrates emerging from different ARSs.

### 4.1 ARS editing domains

#### 4.1.1 Alanyl-tRNA synthetase (AlaRS)

AlaRS erroneously synthesizes Ser- and Gly-tRNA^Ala^. The appended editing domain of AlaRS is responsible for clearing these mischarged products (Figure 2A; Beebe et al., 2003). The editing domain relies on the almost universally conserved wobble base pair G3:U70 to recognize tRNA^Ala^ (Beebe et al., 2008). G3:U70 is also indispensable for tRNA aminoacylation by AlaRS (Hou and Schimmel, 1988; McClain and Foss, 1988). Thus, a single base pair defines tRNA^Ala^ aminoacylation and editing. How the aa-tRNA^Ala^ substrate is transferred from the aminoacylation site to the editing domain remains unknown. Channeling the aa-tRNA^Ala^ between the two active sites would require substantial structural rearrangement of AlaRS to bring the editing domain closer to the aminoacylation domain and prevent complete dissociation of the tRNA (Naganuma et al., 2014). The C-Ala domain could facilitate the movement of the tRNA between the two domains (Guo et al., 2009). Alternatively, the editing domain could bind the tRNA after being released from the aminoacylation domain. Biochemical and biophysical characterization and structural studies are needed to determine the molecular mechanism of aa-tRNA selection by the editing domain of AlaRS.

#### 4.1.2 ThrRS

Most ThrRSs encode a dedicated editing domain that deacylates Ser-tRNA^Thr^ produced in the aminoacylation domain (Dock-Bregeon et al., 2000; Beebe et al., 2004; Korencic et al., 2004). The editing domain is located at the N-terminus of ThrRS and exhibits evolutionary differences. Eukaryotic and bacterial ThrRS have a structurally similar editing domain known as the N2 (Figure 2A). In contrast, the archaeal ThrRS possesses an editing domain structurally homologous to D-aminoacyl-tRNA deacylases (DTD) (Dwivedi et al., 2005; Hussain et al., 2006). Notably, while the N2 and DTD-like domains effectively hydrolyze Ser-tRNA^Thr^, they display distinct tRNA selectivity. For example, the N2 editing domain of *E*. *coli* ThrRS indiscriminately deacylates bacterial and archaeal Ser-tRNA^Thr^. In contrast, the DTD-like domain of ThrRS from the archaeon *Methanosarcina mazei* only hydrolyzes archaeal Ser-tRNA^Thr^ (Beebe et al., 2004). Similarly, the editing domain of *Pyrococcus abyssi* ThrRS was shown to recognize Ser-tRNA^Thr^ while discriminating against other Ser-tRNA substrates (Novoa et al., 2015). These observations suggest that the tRNA specificity of the archaeal ThrRS editing domain may rely on the identity of position 73 (Beebe et al., 2004; Novoa et al., 2015), a conserved U73 in archaeal tRNA^Thr^. In contrast, the same position is variable in bacterial and eukaryotic tRNA^Thr^, consisting of A73 or U73 (Lin et al., 2019). Therefore, the N2 domain may have evolved a relaxed specificity that enables deacylation of tRNA^Thr^ with U73 and A73. This relaxed specificity toward N73 is also observed in the aminoacylation of bacterial and eukaryotic tRNA^Thr^ (Hasegawa et al., 1992; Nameki, 1995). In archaea, the role of N73 in aminoacylation is species-specific, with some species lacking N73 specificity (e.g., *Haloferax volcanii*) and others (e.g., *Aeropyrum pernix*) strongly depending on U73 (Ishikura et al., 2000; Nagaoka et al., 2002). Consequently, a weak correlation exists between editing and aminoacylation of tRNA^Thr^ in the context of N73. In contrast to N73, the anticodon bases play a more important and conserved role in tRNA^Thr^ aminoacylation (Giege and Eriani, 2023). Although direct evidence of the importance of the anticodon bases in editing is not available, a model based on *E*. *coli* ThrRS suggests that tRNA^Thr^ is held by the ThrRS anticodon binding domain, facilitating the CCA-end repositioning from the aminoacylation site to the editing domain (Dock-Bregeon et al., 2004). Whether the DTD-like editing domain of archaeal ThrRS uses a similar mechanism and how it recognizes the U73 is unknown.

#### 4.1.3 Phenylalanyl-tRNA synthetase (PheRS)

The editing activity of PheRS resides in the B3/B4 domain of the β-subunit of the enzyme’s heterodimer (Figure 2A). The B3/B4 domain clears aminoacylation errors involving Tyr and *meta*-Tyr (Roy et al., 2004; Bullwinkle et al., 2014b). This activity of PheRS is essential for preventing mistranslation of Phe codons and maintaining cellular homeostasis. While a detailed investigation of its tRNA specificity is missing, the activity of the PheRS editing domain is affected by changes in the anticodon, as demonstrated by the lack of deacylation of a tRNA^Phe^ G34A mutant (Ling et al., 2009b). Because G34 is an essential element for aminoacylation (Peterson and Uhlenbeck, 1992; Ling et al., 2009b), this result supports a 3′-end translocation model similar to ThrRS N2 editing, in which the anticodon binding domain provides indirect specificity to the editing by holding the tRNA and enabling the transfer of the 3′-end from the aminoacylation site to the editing site (Roy et al., 2004). Whether elements in the acceptor stem or other tRNA regions are directly recognized by the B3/B4 domain of PheRS requires further investigation.

#### 4.1.4 ProRS

ProRS exists in different structural isoforms. In bacteria, the predominant ProRS isoform encodes an editing domain known as the insertion (INS) domain (Figure 2A). The INS domain catalyzes the deacylation of Ala-tRNA^Pro^, which is incorrectly synthesized in the aminoacylation domain of ProRS. To avoid deacylation of cognate Ala-tRNA^Ala^, the INS domain relies on the anticodon binding domain (ABD) of ProRS. The ABD offers specificity by interacting with the unique tRNA^Pro^ anticodon bases G35 and G36 (Das et al., 2014). These bases also serve as identity elements for aminoacylation (Liu et al., 1995; Stehlin et al., 1998). Changes in the identity of these bases prevent the binding of ProRS to the tRNA, impeding tRNA aminoacylation and deacylation. In contrast, mutations in the acceptor stem of tRNA^Pro^ are inconsequential for the catalysis of the INS domain. The role of the anticodon sequence in ProRS editing is further supported by the deacylation of Ala-tRNA^Ala^ mutants with a Pro UGG anticodon (Das et al., 2014). The dependency of the INS domain on the anticodon bases suggests that the ProRS ABD anchors the tRNA, enabling the translocation of the tRNA’s 3′-CCA end for editing. However, the molecular basis of this process remains poorly understood.

#### 4.1.5 IleRS, LeuRS, and ValRS

IleRS, leucyl-tRNA synthetase (LeuRS), and valyl-tRNA synthetase (ValRS) share an evolutionarily related editing domain called CP1 (connecting peptide 1) (Figure 2A). However, the aa-tRNA specificity of each CP1 corresponds to the amino acid(s) mischarged by each ARS. IleRS’s CP1 catalyzes Val- and Cys-tRNA deacylation, whereas LeuRS’s editing domain hydrolyzes Ile- and Nva (norvaline)-tRNA, and ValRS edits Thr- and Abu (α-aminobutyrate)-tRNA (Baldwin and Berg, 1966; Englisch et al., 1986; Lin et al., 1996; Döring et al., 2001; Mursinna et al., 2004; Cvetesic et al., 2014). In addition to their different CP1 substrate specificities, these ARSs use distinct selection strategies for tRNA aminoacylation. IleRS and ValRS rely on anticodon bases and position 73, while LeuRS uses A73 and the unique long variable stem-loop of tRNA^Leu^ (Giege and Eriani, 2023).

For editing by ValRS’s CP1, A73, A35, and C36 are crucial, while other elements like the U4:A69, the anticodon stem U29:A41 base pair, and the core nucleotide G45 moderately contribute to editing (Tardif and Horowitz, 2002). The ValRS CP1’s reliance on the anticodon bases suggests that the ABD facilitates the CCA-end translocation between the aminoacylation and editing sites. The ValRS-tRNA complex supports this model (Fukai et al., 2000). Similarly, some overlap between elements for aminoacylation and editing has been established for LeuRS, albeit with antagonistic evidence emerging from two bacterial LeuRS models. For *E*. *coli* LeuRS, the interaction between G19 in the D-loop and C56 in the T-loop serves as a critical element for aminoacylation and editing (Du and Wang, 2003). However, LeuRS from *Aquifex aeolicus*, a deep-branching bacterium, may lack robust tRNA specificity for editing as it effectively edits Thr, Val, and Ile from different tRNA substrates (Zhu et al., 2007). Nonetheless, the anticodon stem-loop may contribute to transferring the tRNA acceptor stem from the aminoacylation to the editing site, as a mutation of A35 in tRNA^Leu^ mildly decreases editing (Yao et al., 2008). Structural evidence of LeuRS suggests that the anticodon binding domain holds the tRNA in place while the CCA-end moves from the aminoacylation state to the CP1 domain (Tukalo et al., 2005; Palencia et al., 2012). However, how changes in the tRNA anticodon influence LeuRS editing activity remains unclear.

Unlike ValRS and LeuRS, IleRS editing requires nucleotides that are different from those needed for aminoacylation. Nucleotides 16, 20, and 21 in the D-loop are the principal features that facilitate editing by *E*. *coli* IleRS CP1 (Hale et al., 1997). However, a mutant tRNA^Ile^ G16C/Δ20/U21G tRNA^Ile^ is deacylated with similar efficiency as wild-type (Farrow et al., 1999). These discrepancies suggest that D-loop bases influence the transfer of the tRNA but not the chemical step of deacylation (Farrow et al., 1999; Nomanbhoy et al., 1999). Notably, the crystal structure of IleRS bound to the tRNA in an editing conformation did not reveal direct interactions between IleRS and the tRNA D-loop (Silvian et al., 1999). Thus, additional biochemical and structural insights are needed to clarify the tRNA specificity of the IleRS CP1 domain, and how the aa-tRNA^Ile^ traffics between the two IleRS active sites is unknown. This could explain if a direct role of identity elements in editing exists.

### 4.2 *Trans-* editing domains

In contrast to ARSs, *trans*-editing domains generally lack dedicated RNA binding domains (Figure 2B). Nonetheless, several of these enzyme families have developed tRNA specificities based on recognizing tRNA acceptor stem elements. This recognition may be mediated in the same catalytic domain.

#### 4.2.1 INS superfamily

In addition to the INS domain of ProRS, the INS superfamily groups eight families of *trans*-editing domains, YbaK, ProXp-ala, ProXp-x, ProXp-ST1, ProXp-ST2, ProXp-7, ProXp-8, and ProXp-9 (Vargas-Rodriguez and Musier-Forsyth, 2013; Kuzmishin Nagy et al., 2020). Most INS superfamily members are found in bacteria, but each family’s phylogenetic distribution pattern is unique. For example, ProXp-ala is found in all domains of life, whereas YbaK is present only in bacteria. Except for the INS domain, INS superfamily members are single-domain proteins. Interestingly, while these enzymes share high structural homologies and active site features, they display a wider range of aa-tRNA specificities catalyzed by several aaRSs. These deacylases also display distinct mechanisms of substrate selection, including tRNA recognition. In the following subsections, each family’s activities and tRNA specificities are described, except for ProXp-7, ProXp-8, and ProXp-9, whose functions remain unknown (Kuzmishin Nagy et al., 2020).

##### 4.2.1.1 YbaK

YbaK is responsible for the deacylation of Cys-tRNA^Pro^ produced by ProRS (Ahel et al., 2002; An and Musier-Forsyth, 2004; Ruan and Söll, 2005). YbaK uses thiol-specific chemistry for Cys recognition and catalysis (Kumar et al., 2013). However, YbaK lacks robust tRNA selectivity, which results in the deacylation of Cys-tRNA^Cys^ *in vitro* (An and Musier-Forsyth, 2005; Ruan and Söll, 2005; Das et al., 2014; Chen et al., 2019). In a cellular context, YbaK may gain indirect substrate specificity by forming a YbaK-tRNA-ProRS ternary complex that allows shuttling of Cys-tRNA^Pro^ from ProRS to YbaK, avoiding interaction with Cys-tRNA^Cys^ (An and Musier-Forsyth, 2005; Chen et al., 2019). Additionally, the elongation factor Tu protects Cys-tRNA^Cys^ from YbaK but not Cys-tRNA^Pro^. How Cys-tRNA^Pro^ transitions from ProRS to YbaK is unknown.

##### 4.2.1.2 ProXp-ala

ProXp-ala shares the same activity with the ProRS INS domain (Ahel et al., 2003; Vargas-Rodriguez and Musier-Forsyth, 2013). However, unlike the INS domain, ProXp-ala has a robust selectivity for tRNA^Pro^ based on the acceptor stem bases N72 and N73, which corresponds to G72 and A73 in bacteria and C72 and C73 in eukaryotes (Vargas-Rodriguez and Musier-Forsyth, 2013; Das et al., 2014; Ma et al., 2023). ProXp-ala’s specificity prevents cross-reaction with Ala-tRNA^Ala^. Remarkably, ProXp-ala retained its tRNA^Pro^ specificity during evolution from bacteria to eukaryotes, adapting to changes in the identity of the N72 and N73 bases (Vargas-Rodriguez et al., 2020). ProXp-ala is also found fused to the N-terminus of ProRS (lacking an INS domain) in lower eukaryotes from the *Stramenopila*, *Aveolates*, and *Rhizaria* supergroups and the *Leishmania* and *Trypanosoma* genera (Ahel et al., 2003; Vargas-Rodriguez et al., 2020; Parrot et al., 2021). Evidence suggests that the ProRS-fused ProXp-ala can discriminate against Ala-tRNA^Ala^ (Figure 2B; Ahel et al., 2003). In plants, ProXp-ala contains a unique C-terminal domain (CTD) that contributes to the enzyme’s tRNA binding affinity (Figure 2B; Byun et al., 2022). However, the mechanism of substrate selection still needs to be determined for the ProXp-ala-ProRS fusion and plant ProXp-ala.

##### 4.2.1.3 ProXp-x

ProXp-x deacylates tRNAs charged with the non-proteinogenic amino acid Abu, and to a lesser extent, Ala-tRNA^Pro^ (Bacusmo et al., 2018). ProXp-x prefers tRNA substrates carrying an A73, allowing it to recognize different Abu-tRNA substrates. This characteristic of ProXp-x is critical because ProRS, ValRS, LeuRS, and IleRS mischarge Abu (Döring et al., 2001; Nangle et al., 2002; Cvetesic et al., 2014; Bacusmo et al., 2018). Therefore, ProXp-x prevents broad mistranslation of the genetic code with Abu.

##### 4.2.1.4 ProXp-ST1 and ProXp-ST2

ProXp-ST1 and ProXp-ST2 are homologous deacylases that catalyze the hydrolysis of Ser- and Thr-tRNAs (Liu et al., 2015). Both enzymes display broad tRNA specificity, recognizing diverse tRNAs, including tRNA^Val^, tRNA^Ile^, tRNA^Thr^, tRNA^Ala^, and tRNA^Lys^, all of which are mischarged with either Ser or Thr by the corresponding ARS (Jakubowski, 2012; Liu et al., 2015). Thus, the broad tRNA specificity of ProXp-ST1 and ProXp-ST2 prevents mistranslation caused by Ser and Thr mischarging. Despite their overlapping substrate specificities, only ProXp-ST2 has developed direct tRNA recognition based on A73. This bias for tRNAs with A73 prevents hydrolysis of Ser-tRNA^Ser^ due to the G73 of tRNA^Ser^ (Liu et al., 2015). ProXp-ST1 is indifferent to the identity of N73, but whether it hydrolyzes Ser-tRNA^Ser^ is unknown. Because tRNA^Thr^ has an A73, ProXp-ST1 and ProXp-ST2 can efficiently hydrolyze Thr-tRNA^Thr^ *in vitro*. However, ThrRS effectively prevents Thr-tRNA^Thr^ from both enzymes, offering a mechanism that protects correctly aminoacylated tRNA^Thr^ (Liu et al., 2015). A ProXp-ST1-related deacylase, FthB, that hydrolyzes fluorothreonyl-tRNA^Thr^ also exists, but little is known about its tRNA specificity (McMurry and Chang, 2017).

#### 4.2.2 AlaXp

Like the AlaRS editing domain, AlaXp hydrolyzes Ser- and Gly-tRNA^Ala^ (Ahel et al., 2003; Sokabe et al., 2005; Fukunaga and Yokoyama, 2007; Beebe et al., 2008; Chong et al., 2008). AlaXp and the editing domain of AlaRS share high structural and sequence homology and possibly emerged from a common ancestor (Sokabe et al., 2005; Fukunaga and Yokoyama, 2007; Guo et al., 2009). AlaXp exists in three distinct isoforms classified based on their sequence length (Beebe et al., 2008; Novoa et al., 2015). While AlaXp-L and AlaXp-M are functionally identical, AlaXp-S only hydrolyzes Ser-tRNA^Ala^ (Sokabe et al., 2005). Moreover, AlaXp-L and AlaXp-M exhibit tRNA selectivity, achieved via recognition of the G3:U70 base pair that defines the identity of tRNA^Ala^ (Beebe et al., 2008). In contrast, AlaXp-S lacks tRNA specificity (Novoa et al., 2015). AlaXp-S is considered an ancestral version of the AlaXp family. Thus, AlaXp may have been a general aa-tRNA deacylase that gradually evolved tRNA specificity. A single Arg residue may determine the tRNA specificity of AlaXp (Novoa et al., 2015).

#### 4.2.3 D-aminoacyl-tRNA deacylase (DTD)

DTDs prevent the cellular accumulation of D-aa-tRNAs stemming from several ARSs (Calendar and Berg, 1967; Soutourina et al., 2000). Three distinct DTD isoforms are found in organisms from all domains of life: DTD1 in most bacteria and eukaryotes, DTD2 in plants and archaea, and DTD3 in cyanobacteria (Kumar et al., 2022). Bacterial DTD requires a purine (A/G) in position 73 for effective aa-tRNA deacylation (Kuncha et al., 2018b). The specificity of bacterial DTD enables deacylation of several tRNA substrates while preventing deacylation of Gly-tRNA^Gly^, which has a conserved U73 in bacteria (Routh et al., 2016). Interestingly, N73 evolved from U to A73 in cytosolic tRNA^Gly^. This change in the identity of N73 prompted a switch in the tRNA specificity of eukaryotic DTD1, which prefers pyrimidine instead of purine (Gogoi et al., 2022). Whether the identity of N73 plays a role in the deacylation of D-aa-tRNAs is yet to be determined.

In addition to D-aa-tRNAs, bacterial DTD can inherently deacylate the achiral Gly from tRNA^Ala^ (Pawar et al., 2017). Bacterial DTD selects tRNA^Ala^ based on the G3:U70 and A73, which are essential for tRNA^Ala^ aminoacylation by AlaRS (Hou and Schimmel, 1988; McClain and Foss, 1988; Pawar et al., 2017). Finally, the Animalia-specific tRNA deacylase (ATD), a DTD paralog that hydrolyzes Ala-tRNA^Thr^ synthesized by AlaRS, may use G4:U69 and U73 for tRNA selection. The G4:U69 of tRNA^Thr^ enables mischarging by AlaRS (Sun et al., 2016; Kuncha et al., 2018a).

## 5 Outlook

Despite the strong correlation between the role of identity elements in tRNA editing and aminoacylation, our overall knowledge is limited. The tRNA specificities of several editing enzymes are unknown or poorly understood. For example, whether the B3/B4 domain of PheRS relies on tRNA acceptor stem is still unknown. The lack of molecular tools to prepare aa-tRNA substrates has significantly contributed to our poor understanding of the relationship between identity elements and editing. Producing mischarged tRNA variants using ARSs is challenging because mutating identity elements results in poor aminoacylation. Most available data for the tRNA specificity determination of CP1 domains are based on ATP consumption assays (Farrow et al., 1999; Tardif and Horowitz, 2002; Du and Wang, 2003; Zhu et al., 2007). This method integrates the effect of tRNA mutations in aminoacylation and editing. Thus, establishing the direct contribution of tRNA elements to editing can be intricate because the same elements can impact aminoacylation. The development of flexizyme technology now offers a powerful tool to investigate the role of identity elements in aa-tRNA editing (Murakami et al., 2006). This catalytic RNA ligates virtually any amino acid to tRNAs regardless of their sequence. Thus, it enables the preparation of diverse aa-tRNA mutant substrates to examine identity elements in the context of editing comprehensively (Das et al., 2014; Liu et al., 2015; Novoa et al., 2015; Danhart et al., 2017; Vargas-Rodriguez et al., 2020; Watkins et al., 2024). Adopting flexizyme can help establish and clarify the substrate specificities of many *cis*- and *trans*-editing enzymes from diverse species and across domains of life. Ultimately, this will expand our understanding of the dual role of identity elements in editing and aminoacylation, which, in turn, can provide novel insights into the contribution of editing enzymes to the establishment of the genetic code (Beebe et al., 2003).

## Author contributions

EC: Writing – review and editing, Writing – original draft. OV-R: Writing – review and editing, Writing – original draft.
